# Complementary N Uptake Strategies between Tree Species in Tropical Rainforest

**DOI:** 10.1155/2014/427194

**Published:** 2014-10-29

**Authors:** J. C. Roggy, H. Schimann, D. Sabatier, J. F. Molino, V. Freycon, Anne-Marie Domenach

**Affiliations:** ^1^INRA, UMR Ecologie des Forêts de Guyane, Campus Agronomique, BP 709, 97387 Kourou Cedex, French Guiana; ^2^UMR Botanique et Bioinformatique de l'Architecture des Plantes (AMAP), TA A51/ PS2, 34398 Montpellier Cedex 5, France; ^3^UR Dynamique des Forets Naturelles, TA C-37/D, Campus International de Baillarguet, 34398 Montpellier Cedex 5, France; ^4^Solicaz, c/o Guyane Technopole, 16 bis rue du 14 Juillet, 97 300 Cayenne, French Guiana

## Abstract

Within tree communities, the differential use of soil N mineral resources, a key factor in ecosystem functioning, may reflect functional complementarity, a major mechanism that could explain species coexistence in tropical rainforests. *Eperua falcata* and *Dicorynia guianensis*, two abundant species cooccurring in rainforests of French Guiana, were chosen as representative of two functional groups with complementary N uptake strategies (contrasting leaf *δ* ^15^N signatures related to the *δ* ^15^N of their soil N source, NO_3_^−^ or NH_4_^+^). The objectives were to investigate if these strategies occurred under contrasted soil N resources in sites with distinct geological substrates representative of the coastal rainforests. Results showed that species displayed contrasting leaf *δ* ^15^N signatures on both substrates, confirming their complementary N uptake strategy. Consequently, their leaf ^15^N can be used to trace the presence of inorganic N-forms in soils (NH_4_^+^ and NO_3_^−^) and thus to indicate the capacity of soils to provide each of these two N sources to the plant community.

## 1. Introduction

How species diversity is maintained in tropical rainforests is a critical issue in contemporary ecology. According to the niche complementarity hypothesis, coexisting plants are thought to complement each other in traits related to resource foraging. Consequently, resource partitioning and functional complementarity could be important in maintaining species diversity in plant communities. Mineral nitrogen is a key factor in ecosystem functioning. Within tree communities, the differential use of soil N mineral resources (nitrate, ammonium) may, among other mechanisms, reflect tree functional complementarity [1–3]. The spatial variability of soil nitrate-to-ammonium ratio may partly result from the spatially heterogeneous occurrence of two of the major soil microbial processes, nitrification and denitrification [4]. A number of studies have linked the N uptake strategies of tropical trees to their successional status (see [5] for review). In contrast, Houlton et al. [6] and Russo et al. [7] showed that coexistence among functionally diverse species was not linked to the particular form of N available. In French Guiana, Roggy et al. [2] reported that among non-N_2_-fixing trees two groups of late-successional species with contrasting natural leaf ^15^N abundance coexisted. This was interpreted as different abilities of species to utilize different soil mineral N pools. Based on these results, we carried out a preliminary study in French Guiana with two abundant cooccurring late-successional species,* Eperua falcata* and* Dicorynia guianensis* [8]. These species were chosen as representative of the two groups of non-N_2_-fixing species with contrasting leaf *δ* ^15^N signatures. We demonstrated that both species exhibited complementary N uptake strategies and that their leaf *δ* ^15^N were related to the *δ* ^15^N signatures of their soil N source (NO_3_^−^ or NH_4_^+^). In this study, the objectives were to investigate if complementary N uptake strategies of these two tree species occurred under contrasted soil N resources in sites with distinct geological substrates representative of the coastal rainforest in French Guiana. Specifically, we tested the occurrence of different leaf *δ* ^15^N signatures associated with the two species on volcanic-plutonic rocks of the Paramaca series (high total soil nitrogen concentration) and schists of the Orapu series (low total soil nitrogen concentration). We also estimated variations in nitrification and denitrification potentials in soils and litters under the two species.

## 2. Materials and Methods

### 2.1. Study Sites

**Figure 1:**
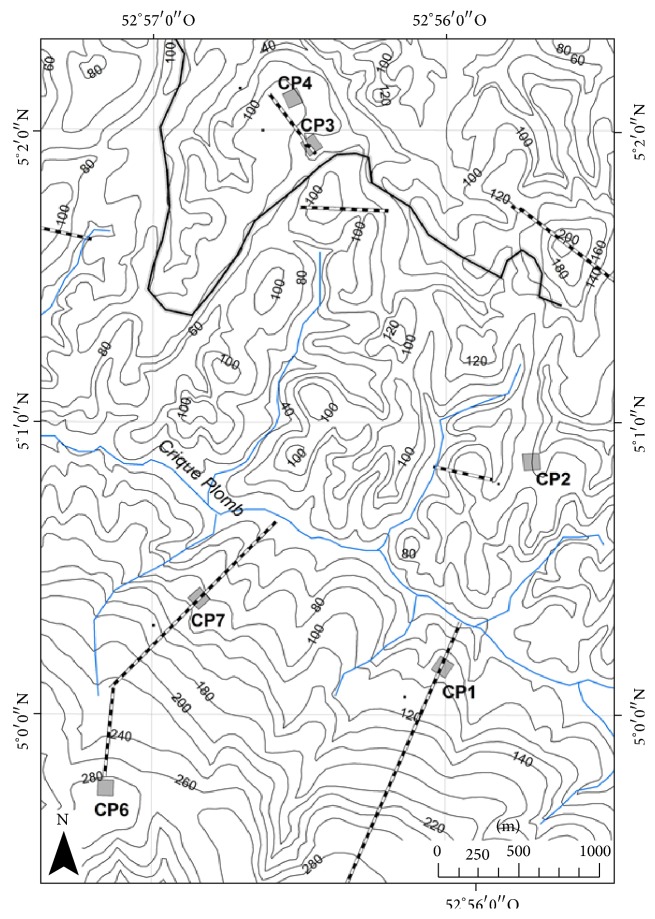
Map of the 6 1-ha plots studied in natural rainforests developed on two geomorphologic domains in French Guiana: volcanic-plutonic domain (CP1, CP6, and CP7) and schist domain (CP2, CP3, and CP4). The small river (Crique Plomb) running along a geological boundary divides the area into two distinct geomorphologic zones.

The study was conducted in Crique Plomb, an experimental site of natural forest in the northern part of French Guiana (5°0′-5°2′N, 52°56′-52°57′W). This site is characterized by a clear segregation, by a small river (Crique Plomb) running along a geological boundary, between two geomorphologic domains representative of the contrast between metamorphosed sediments and a volcanic formation (Figure 1). The northern part lies in a hilly area where the geologic substrate is pelitic schist of the Precambrian “flyschoid series” (Orapu schist of Armina series [9, 10]). Physical and chemical erosion of a preexisting deep oxisol have resulted in a deep oxisol with a deep vertical drainage. The southern part is a single topographic unit where the geologic substrates are of volcanic-plutonic origins (Paramaca series). The soil cover units consist in numerous zones of ferricrete interspersed with deep nonhydromorphic soils.

Six plots of one hectare were selected in the two main geomorphologic domains, that is, three replicates per domain: plots CP3, CP2, and CP4 in the schist domain and plots CP1, CP7, and CP6 in the volcanic-plutonic domain. On each domain, plots were separated from at least 1000 m in order to integrate potential spatial variations in nutrient uptake by trees (Figure 1).

Although the two soil types had similar pH levels, chemical analyses showed significant differences (Table 1). The soils of the volcanic-plutonic domain had higher levels of nitrogen (0.83% versus 0.51%) and carbon (13.58% versus 8.61%) but identical C-to-N ratios (≈16).

Three angiosperm families represent around 60% of individuals in the Crique Plomb area: the Lecythidaceae, Caesalpinioideae, and Euphorbiaceae. More than 700 tree species with stems over 10 cm DBH (diameter at breast height ~ 1.30 m) belonging to 270 genera and 68 families were recorded for 12,000 inventoried individuals. There are around 150 tree species per hectare in the plots and rare species (species with few individuals) dominate.

The climate is characterized by a clear seasonal pattern: a wet season from December to July, which is normally interrupted in February or March by a short dry period, and a long dry season from August to November with monthly precipitation of less than 100 mm. Average annual precipitation is around 3000 mm. Mean temperature is 25°C with low seasonal changes [11]. All measurements were taken at the end of the rainy season.

### 2.2. Tree Species, Soils, and Litter Sampling

*Dicorynia guianensis* Amsh. (tribe Cassieae) and* Eperua falcata* Aublet (tribe Detarieae) are two cooccurring, shade hemitolerant* Caesalpinioideae* species belonging to the late-successional species group [12]. Bothare known as non-N_2_-fixing species with high and low leaf *δ* ^15^N values, respectively [1, 2]. Both exhibit a Paris-type mycorrhizal association [13, 14]. In each of the six plots, at least five trees per species (representing up to 25% to 100% of the individuals per species per plot) with a DBH of 10 cm or more were randomly selected by pairs of the two species. Leaves from the upper canopy were collected using a shotgun. Only young, fully expanded leaves were selected for analysis since *δ* ^15^N might be sensitive to leaf age [15]. Three samples of litter and soil were collected underneath the canopy of each individual tree. Leaves were analyzed for *δ* ^15^N and total nitrogen. Soil and litter samples were analyzed for their capacities (i.e., potential rates) to express nitrification and denitrification (both processes being directly involved in the balance between NH_4_^+^ and NO_3_^−^). Soils were analyzed for NO_3_^−^.

We will focus on potential activities rather than on actual activities because they are less fluctuating and less sensitive to short spatiotemporal variations, which allow between sites comparisons. Moreover, potential activities are not under the control of limitation or inhibition factors of the environment and represent the maximal capacity of the enzyme pool in soil to realize a biotransformation [16].

### 2.3. *δ* ^15^N Analyses

The leaf laminae (i.e., without petioles or rachis) were oven-dried at 60°C for 48 h. Leaf laminae were then milled to a fine powder and packed in airtight containers. Nitrogen isotope ratios (^15^N/^14^N) and total nitrogen concentrations were measured as described by Casabianca [17], using an elemental analyzer (SCA, CNRS, Solaize, France) coupled with a mass spectrometer (Finnigan Mat, DS, Bremen, Germany). The precision of measurement was 0.2‰ (standard error of the mean, *n* = 1000). Results are reported as percent N (leaf N (%)) and *δ* ^15^N (‰) where

(1) δ15N(‰)=(Rsample−Rstandard)Rstandard×1000,

where the standard is atmospheric N_2_ (atom%^15^N = 0.3663, [18]).

### 2.4. Denitrifying Enzymatic Activity (DEA) in Soil and Litter

The enzymatic potential of denitrification was measured according to Lensi et al. [19]. For each sample, 10 g of sieved and air-dried soil or 2 g of air-dried litter was placed in a 150 mL plasma-flask and sealed with rubber stoppers. In each flask, air was removed with a vacuum-pump and replaced with a He–C_2_H_2_ mixture (90/10, vol/vol) to inhibit the N_2_O-reductase. A solution containing 100 *μ*g of N-KNO_3_, 1 mg of C-glucose, and 1 mg of C-glutamic acid/g of sample (soil or litter) was added to ensure saturation. The flasks were incubated at 28°C for 5 h and gas samples of 200 *μ*L were analyzed for N_2_O on a gas chromatograph with an electron capture detector (VARIAN 3800-CP, Les Ulis, France). The reason for these short incubations was to avoid* de novo* enzymatic synthesis and cell growth. DEA was expressed in ng of N-N_2_O/g/h.

### 2.5. Nitrate in Soil and Nitrifying Enzymatic Activity (NEA) in Soil and Litter

The enzymatic potential of nitrification was measured according to Lensi et al. [20]. Each sample was divided into 20 g (soil) or 2 g (litter) subsamples and placed in 150 mL plasma flasks. The first subsamples were used to estimate the initial NO_3_^−^ content expressed in *μ*g N-NO_3_^−^/g soil or litter. They were supplied with 6 cm^3^ of a suspension of a denitrifying organism (*Pseudomonas fluorescens*, O.D._580_ = 2) in a solution containing glucose and glutamic acid (1 mg C/g dry soil or litter of final C content for each compound). Soils and litter were incubated for 24 h in anaerobiosis to ensure total transformation of the nitrate into N_2_O. Anaerobic conditions and N_2_O-reductase inhibition were ensured by replacing the atmosphere of each flask with a He–C_2_H_2_ mixture (90/10, vol/vol). N_2_O accumulation was monitored until total conversion of NO_3_^−^ into N_2_O (i.e., a constant value). The second subsamples were used to determine the kinetics of NO_3_^−^ accumulation in soil and litter. After enrichment with 2 cm^3^ of a (NH_4_)_2_SO_4_ solution (final N content 0.2 mg/g dry soil or litter and 80% W.H.C), the flasks were sealed with Parafilm^©^ and incubated at 28°C for 48 h in a horizontal position to ensure optimal, homogeneous aeration of the sample. Samples were then enriched with 4 cm^3^ of a* P. fluorescens *suspension (O.D._580_ = 2) in a glucose/glutamic acid solution (with concentration values adjusted to achieve 1 mg C/g). Then anaerobiosis and N_2_O inhibition were obtained in the flasks as described above and the N_2_O accumulation was monitored until a constant value was reached. The enzymatic potential of nitrification was computed by subtracting the nitrate initially present in the sample (soil or litter) from the nitrate accumulated after aerobic incubation and expressed in ng of N-NO_3_/g/h.

### 2.6. Statistical Analysis

All analyses were made using the Statistica 7.1 software (StatSoft Inc., 1984–2007). Data were ranked to avoid the assumptions of normality [21], and the differences between the means were analysed with the Mann-Whitney *U*-test (*P* < 0.05) after a one-way ANOVA of Kruskal-Wallis.

## 3. Results and Discussion

**Figure 2:**
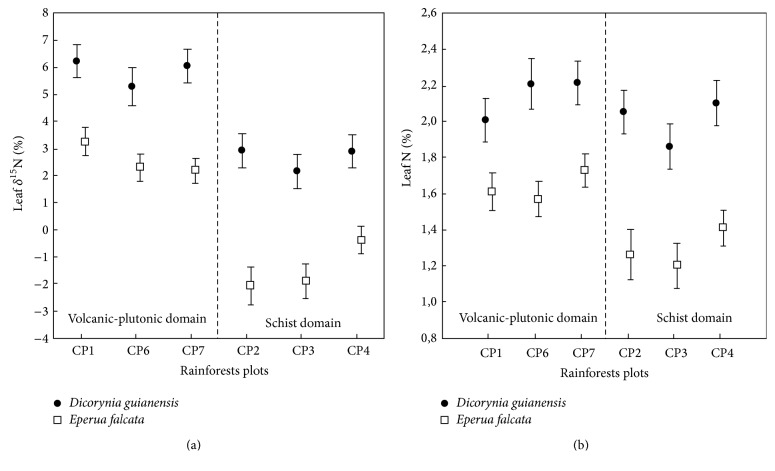
(a) Leaf natural N^15^abundance (*δ* ^15^N ‰) and (b) total leaf nitrogen content (%N) in* Dicorynia guianensis* and* Eperua falcata* in rainforest plots developed on two geomorphologic domains in French Guiana. Six one-ha plots: volcanic-plutonic domain (CP1, CP6, and CP7) and schist domain (CP2, CP3, and CP4) (means ± SE).

Leaf samples of* D. guianensis* and* E. falcata* had consistently different ^15^N isotopic signatures (average difference between the two species around 3.5‰, Figure 2), with lower values on schist than on the volcanic-plutonic domain. A similar magnitude of difference between these values was observed on both substrates. This variability of the *δ* ^15^N between species may be due to spatial variability of soil *δ* ^15^N, which essentially reflect the isotopic signatures of organic pools [22]. In French Guiana, the *δ* ^15^N of the organic matter in soils and litters vary among forests on different substrates but remain spatially uniform for a given type of soil [12, 23] and in litters and soils collected underneath* E. falcata* and* D. guianensis* [8]. These studies showed that the inorganic N assimilated by these trees came from an organic N source with a homogeneous isotopic composition. A second source of variability could come from different mycorrhizal status of trees, mycorrhized trees having access to organic soil N with a particular ^15^N abundance [22]. In French Guiana, both species exhibit a Paris-type mycorrhizal association [14], which exclude differentiation of leaf *δ* ^15^N due to different mycorrhizal status. A third source of variability could lie in abilities of roots to use various pools of soil inorganic N with different ^15^N abundances. Thus, the foliar *δ* ^15^N observed in the two species would be linked to the *δ* ^15^N of the inorganic N sources they used [24], knowing that uptake by and translocation of N within the plants are not considered to cause appreciable isotopic fractionation [7]. In this study, the differences of *δ* ^15^N observed in leaves of* E. falcata* and* D. guianensis* showed clearly that both species used different soil inorganic N sources with different *δ* ^15^N signatures on both substrates. Differences of *δ* ^15^N between the two soil N sources are due to fractionation of ^15^N during enzyme-driven transformation process (e.g., nitrification [22]).

Soil microbial activities were spatially partitioned between the soil (denitrification) and litter (nitrification, Table 2), confirming the results found by Schimann et al. [8] on a different type of substrate. Despite different total soil C and N concentrations in the two substrates (Table 1), soil nitrate contents and microbial activities were similar (Table 2). Previous studies (Roggy et al. pers com) have showed that N_2_ fixation by trees is twice higher on schist than on volcanic-plutonic soil (6.5 versus 3.5 kg N/ha, resp.). Additional inputs of labile organic matter by litters of N_2_-fixing trees are known to enhance microbial growth and activities [25, 26] and particularly nitrification rates [4, 27]. Thus, nitrification is expressed at a similar level on both substrates. Nevertheless, these processes are not sufficient to allow accumulating organic matter in soils as shown by the lower contents of C and N in schist soil than in volcanic-plutonic soil. As indicated, the two species displayed different isotopic signatures whatever the geological substrates but the *δ* ^15^N values were lower in the schist soils (Figure 2). In these soils, there is probably a global decrease of *δ* ^15^N in litters and soils due to the higher proportion of N_2_-fixing species in these forests which supply litters with low *δ* ^15^N values [1, 23, 28]. On this type of substrate, NH_4_^+^ and NO_3_^−^ are supposed to be produced from a more ^15^N-depleted organic matter as compared to the organic matter of the volcanic-plutonic soil.

## 4. Conclusions

In conclusion, our results together with those of Schimann et al. [8] showed that the two tree species exhibit complementary N uptake strategies on all substrates representatives of coastal rainforests of French Guiana with contrasted soil N resources. Consequently, their leaf *δ* ^15^N can be used to trace the presence of inorganic N-forms in soils (NH_4_^+^ and NO_3_^−^) and thus to indicate the capacity of soils to provide each of these two N sources to the plant community.

## Figures and Tables

**Table 1:** Soil characteristics in 6 one-ha plots.

	*n*	pH	%N	%C	C : N
Volcanic-plutonic domain					
CP1		4.89 (0.19)	0.79 (0.06)	12.55 (1.14)	15.90
CP6		5.00 (0.08)	0.73 (0.05)	11.52 (0.90)	15.70
CP7		5.13 (0.17)	0.94 (0.07)	16.46 (1.60)	17.42
Mean	**32**	**5.00 (0.08)**	**0.83 **(0.04)^*^	**13.58 **(0.81)^*^	**16.44 (0.94)**
Schist domain					
CP2		4.98 (0.12)	0.49 (0.07)	7.89 (0.26)	15.93
CP3		5.02 (0.11)	0.46 (0.02)	7.71 (0.41)	16.86
CP4		5.47 (0.37)	0.59 (0.06)	10.17 (1.04)	17.24
Mean	**32**	**5.13 (0.12)**	**0.51 **(0.02)^*^	**8. 61 **(0.44)^*^	**16.72 (0.67)**

Soil characteristics (pH, C : N, N, and C concentration) in 6 one-ha plots (CP2, CP3, and CP4 and CP1, CP6, and CP7) of rainforest developed on two geomorphologic domains (schist domain and volcanic-plutonic domain) in French Guiana. ^*^Differences are significant at *P* < 0.05 (ANOVA of Kruskal-Wallis with geomorphological domain as main factor).

**Table 2:** Denitrification, nitrification, and nitrate content in soils and litters.

	*n*	Soil DEA ng N-N_2_O/g/h	Litter DEA ng N-N_2_O/g/h	Soil NEA ng N-NO_3_/g/h	Litter NEA ng N-NO_3_/g/h	Soil NO_3_^−^ *μ*g N-NO_3_^−^/g
Volcanic-plutonic domain						
*Dicorynia guianensis *	15	225.04 (65.54)	38.26 (12.56)	0.00 (0.00)	22.86 (16.19)	3.68 (0.81)
*Eperua falcata *	17	247.13 (75.68)	65.41 (14.51)	0.00 (0.00)	28.81 (18.69)	3.03 (0.94)
Mean	**32**	**234.50 (44.19)**	**49.89 **(6.79)^*^	**0.00 (0.00)**	**25.41 (13.74)**	**3.40 (0.53)**
Schist domain						
*Dicorynia guianensis *	15	213.32 (50.74)	15.52 (3.63)	0.00 (0.00)	23.96 (18.07)	2.04 (0.59)
*Eperua falcata *	17	236.47 (52.65)	20.75 (3.77)	0.00 (0.00)	25.58 (18.75)	2.30 (0.61)
Mean	**32**	**224.46 (38.97)**	**18.03 **(6.00)^*^	**0.00 (0.00)**	**24.77 (12.12)**	**2.16 (0.47)**

Denitrification enzyme activity (DEA, ng of N-N_2_O/g/h), nitrification enzyme activity (NEA, ng of N-NO_3_^−^/g/h) in soils and litters, and NO_3_^−^ content (*μ*g N-NO_3_^−^/g soil) in soils beneath *Dicorynia guianensis* and *Eperua falcata* in rainforest developed on two geomorphologic domains (schist domain and volcanic-plutonic domain) in French Guiana (means ± SE). ^*^Differences between means are significant at *P* < 0.05.
